# Sex differences in early dyspnea relief between men and women hospitalized for acute heart failure: insights from the RELAX-AHF study

**DOI:** 10.1007/s00392-016-1051-4

**Published:** 2016-11-12

**Authors:** Sven Meyer, John R. Teerlink, Marco Metra, Piotr Ponikowski, Gad Cotter, Beth A. Davison, G. Michael Felker, Gerasimos Filippatos, Barry H. Greenberg, Tsushung A. Hua, Thomas Severin, Min Qian, Adriaan A. Voors

**Affiliations:** 1 Department of Cardiology, University Medical Center Groningen, University of Groningen, Groningen, The Netherlands; 2 Department of Cardiology, Heart Center Oldenburg, European Medical School Oldenburg-Groningen, Carl von Ossietzky University Oldenburg, Oldenburg, Germany; 3 University of California at San Francisco and San Francisco Veterans Affairs Medical Center, San Francisco, CA USA; 4 Cardiology, Department of Medical and Surgical Specialties, Radiological Sciences, and Public Health, University of Brescia, Brescia, Italy; 5 Medical University, Clinical Military Hospital, Wroclaw, Poland; 6 Momentum Research Inc., Durham, NC USA; 7 Duke University School of Medicine, Duke Heart Center, Durham, NC USA; 8 Athens University Hospital, Attikon, Athens, Greece; 9 University of California at San Diego, La Jolla, CA USA; 10 Novartis Pharmaceuticals Corp., East Hanover, NJ USA; 11 Novartis Pharma AG, Basel, Switzerland; 12 Columbia University Medical Center, New York, NY USA

**Keywords:** Serelaxin, Acute heart failure, Sex, Gender, Dyspnea

## Abstract

**Aims:**

Women with heart failure are typically older, and more often have hypertension and a preserved left ventricular ejection fraction as compared with men. We sought to analyze if these sex differences influence the course and outcome of acute heart failure.

**Methods and results:**

We analyzed sex differences in acute heart failure in 1161 patients enrolled in the RELAX-AHF study. The pre-specified study endpoints were used. At baseline, women (436/1161 patients) were older, had a higher left ventricular ejection fraction, a higher rate of hypertension, and were treated differently from men. Early dyspnea improvement (moderate or marked dyspnea improvement measured by Likert scale during the first 24 h) was greater in women. However, dyspnea improvement over the first 5 days (change from baseline in the visual analog scale area under the curve (VAS AUC) to day 5) was similar between men and women. Women reported greater improvements in general wellbeing by Likert, but no such benefits were evident with the VAS score. Multi-variable predictors of moderate or marked dyspnea improvement were female sex (*p* = 0.0011), lower age (*p* = 0.0026) and lower diuretic dose (*p* = 0.0067). The additional efficacy endpoints of RELAX-AHF were similar between men and women and serelaxin was equally effective in men and women.

**Conclusions:**

Women exhibit better earlier dyspnea relief and improvement in general wellbeing compared with men, even adjusted for age and left ventricular ejection fraction. However, in-hospital and post-discharge clinical outcomes were similar between men and women.

This trial is registered at ClinicalTrials.gov, NCT00520806.

## Introduction

Women and men show marked differences both in the onset of heart failure and in established chronic heart failure [1, 2]. We recently showed that clinical characteristics of men and women admitted for acute heart failure are also different [3]. Relative to men, women typically more often show features such as hypertension, atrial fibrillation and preserved left ventricular ejection fraction, whereas men usually present with ischemic heart disease, history of myocardial infarction, reduced left ventricular ejection fraction, and specific medical and device treatment [3].

In previous studies, women admitted for acute heart failure received lower oral and intravenous diuretic doses, had fewer dose increases, lost less body weight during hospitalization, and had a longer length of hospitalization compared with men [3, 4]. However, no studies have specifically focused on differences in dyspnea relief and changes in general wellbeing between men and women admitted for acute heart failure.

Here, we investigate sex differences in early and persistent dyspnea relief as well as additional efficacy endpoints, and analyzed patient features and heart failure characteristics in men and women hospitalized for acute heart failure enrolled in the RELAX-AHF study [5]. The RELAX-AHF study tested the effects of serelaxin, a recombinant form of the natural hormone human relaxin 2, vs. placebo on dyspnea relief on top of standard of care [5], and provided insights into specific effects of serelaxin in acute heart failure [6, 7].

## Patients and methods

### Study design, population and treatment

The RELAX-AHF study was a multi-center, double-blind, randomized, controlled trial, comparing the intravenous administration of serelaxin for up to 48 h vs. placebo on top of standard of care. Patients were randomized within the initial 16 h of hospital admission for AHF with congestion and dyspnea, additionally having elevated natriuretic peptide levels, mild to moderate renal dysfunction, and systolic blood pressure >125 mmHg. Notably, patient-reported dyspnea improvement was evaluated by the two primary efficacy endpoints of the RELAX-AHF study: first, change from baseline to day 5 in the visual analog scale area under the curve (VAS AUC) and, second, the proportion of patients with moderate or marked dyspnea improvement as indicated by Likert scale ratings at 6, 12 and 24 h (all three), both analyzed by intention to treat [5]. The VAS was a 0–100-mm long scale on which each patient marked the level of dyspnea and the distance from the 0-level of the scale was measured. VAS appropriately allows quantification of within-subject changes of repeated measurements as it has the sensitivity required to measure changes. The 7-item Likert scale is a psychometric instrument for the grading of dyspnea. Patients were asked to rate the degree of improvement in response to therapy within the spectrum of categories ranging from markedly better to markedly worse. Notably, for the patients with available dyspnea Likert assessments, those following death, worsening heart failure or heart failure/renal failure rehospitalization event were imputed as the worst score of the Likert scale as ‘markedly worse’ (worse Likert score = −3); for the “Time to Moderately or Markedly Better Dyspnea Through Day 5” analyses, for patients who died, had worsening heart failure or re-hospitalization due to heart failure, the day was set to 6 days.

### Ethics

The RELAX-AHF study was approved by all local Ethics Committees and complied with the Declaration of Helsinki guidelines. Written informed consent was obtained from all patients.

### Statistical analyses

1161 patients were randomized in RELAX-AHF [the Intent-to-Treat (ITT) population]. For outcomes with complete data, the analysis included all randomized patients (*n* = 1161). For other outcomes, cases with missing outcome data were omitted from the analysis under an assumption of missing at random. Missing values in baseline covariates were imputed using treatment-specific medians for continuous variables and treatment-specific modal values for categorical variables. The criterion for statistical significance is *p* ≤ 0.05 two-tailed for all analyses. Patient baseline characteristics, symptomatic response, diuretic doses, treatment response, and post-discharge outcomes were compared by sex using *t* tests for continuous variables, Chi squared tests or Fisher’s exact tests for categorical variables, and log-rank tests for time-to-event outcomes. Effects of treatment by sex interaction on clinical outcomes were examined using multiple linear regression models for continuous endpoints, logistic regression models for categorical endpoints, and Cox proportional hazards models for time-to-event endpoints. Three-way interaction effects of sex, treatment, and EF status on clinical outcomes were also examined using the same approach. A multi-variable logistic regression model was used to assess the association between moderately or markedly better dyspnea on the Likert scale at 6, 12 and 24 h and selected baseline characteristics, including sex, HFpEF status as defined by ESC-guideline criteria [8], age, pulse pressure, heart rate, and loop diuretic dose. The effect of treatment by sex interaction on markedly or moderately improved dyspnea at each time point (6, 12 and 24 h) was also estimated using logistic regression models. Repeated measures ANOVA models were used to estimate mean changes in biomarkers (hs-troponin-T, NT-proBNP and cystatin-C) from baseline through day 14, mean changes in patient-reported dyspnea according to visual analog scale from baseline to day 5, and mean total daily dose of IV diuretics (mg) from day 1 to day 5, stratified by treatment and sex. A linear regression model was used to estimate the effects of treatment, sex and their interaction on total dose of IV diuretics from day 1 to day 5. Fisher’s exact tests were used to assess the association between treatment and physician-assessed signs and symptoms of congestion at day 2 for each sex. Effects of treatment by sex interaction on physician-assessed signs and symptoms of congestion at day 2 were evaluated using proportional-odds logistic regression models. Kaplan–Meier survival curves for CV mortality through day 180 were generated for all sex and treatment combinations, and compared using log-rank tests. A Cox proportional hazard model was used to examine the treatment effect by sex.

Analyses were performed by the Statistical Analysis Center at Columbia University.

## Results

### Baseline characteristics

Details of baseline patient characteristics are shown in Table 1. The RELAX-AHF study comprised 725 men and 436 women. Women were on average 6 years older, had about 10% higher LVEF and a lower proportion of LVEF <40%, and had less frequent ischemic heart disease or a history of chronic heart failure one month prior. Before hospitalization, women had lower NYHA class symptoms and they more often had hypertension, while less frequently being cigarette smokers or showing peripheral vascular disease, asthma, bronchitis, or COPD, myocardial infarction and history of CRT or ICD procedures and implanted devices. Women less often received oral loop diuretics 30 days before study entry and were more often treated with digoxin. Plasma levels of hemoglobin, creatinine, uric acid, troponin and estimated glomerular filtration rate were lower in women, whereas they had higher levels of total cholesterol. There were no significant sex differences in clinical variables or congestion.

**Table 1:** Baseline characteristics by sex (*n* = 1161)

Variables	Total^a^ (*n* = 1161)	Men^a^ (*n* = 725)	Women^a^ (*n* = 436)	*p* value^b^
Demographics and HF characteristics				
Age (years)	72.0 (11.2)	69.8 (11.7)	75.8 (9.2)	<0.0001 [S]
Serelaxin administration (%)	581 (50.0%)	368 (50.8%)	213 (48.9%)	0.5295 [2]
White	1096 (94.4%)	680 (93.8%)	416 (95.4%)	0.2450 [2]
Geographic region				0.0001 [2]
Eastern EU	562 (48.4%)	315 (43.4%)	247 (56.7%)	
Western EU	204 (17.6%)	144 (19.9%)	60 (13.8%)	
South America	71 (6.1%)	45 (6.2%)	26 (6.0%)	
North America	114 (9.8%)	85 (11.7%)	29 (6.7%)	
Israel	210 (18.1%)	136 (18.8%)	74 (17.0%)	
US-Like^c^	786 (67.7%)	540 (74.5%)	246 (56.4%)	<0.0001 [2]
Left ventricular EF (%)	38.6 (14.6)	35.1 (13.2)	44.7 (14.9)	<0.0001 [S]
EF <40%	598 (54.8%)	446 (64.8%)	152 (37.7%)	<0.0001 [2]
Ischemic heart disease	603 (51.9%)	419 (57.8%)	184 (42.2%)	<0.0001 [2]
Time to randomization (h)	7.9 (4.6)	7.7 (4.8)	8.2 (4.4)	0.0384 [1]
CHF 1 month prior	861 (74.2%)	557 (76.8%)	304 (69.7%)	0.0074 [2]
HF hospitalization past year	397 (34.2%)	260 (35.9%)	137 (31.4%)	0.1225 [2]
NYHA class 30 days before admission				0.0014 [2]
I	323 (28.1%)	186 (25.8%)	137 (31.8%)	
II	304 (26.4%)	174 (24.2%)	130 (30.2%)	
III	389 (33.8%)	268 (37.2%)	121 (28.1%)	
IV	135 (11.7%)	92 (12.8%)	43 (10.0%)	
Clinical variables				
Body mass index (kg/m^2^)	29.3 (5.7)	29.3 (5.3)	29.3 (6.3)	0.8964 [S]
Syst. blood pressure (mmHg)	142.2 (16.6)	141.2 (16.5)	143.8 (16.7)	0.0110 [1]
Diast. blood pressure (mmHg)	79.0 (14.2)	79.8 (14.0)	77.7 (14.5)	0.0125 [1]
Heart rate (beats/min)	79.7 (14.9)	79.1 (14.5)	80.6 (15.6)	0.1093 [1]
Respiratory rate (breaths/min)	21.9 (4.6)	21.7 (4.6)	22.3 (4.6)	0.0299 [1]
Congestion at baseline				
Edema	910 (78.9%)	578 (80.4%)	332 (76.3%)	0.1010 [2]
Orthopnea	1106 (95.8%)	689 (95.8%)	417 (95.9%)	0.9773 [2]
JVP	850 (75.5%)	533 (76.0%)	317 (74.6%)	0.5845 [2]
Dyspnea on exertion	1136 (99.6%)	708 (99.7%)	428 (99.5%)	0.6351 [3]
Dyspnea by VAS	44.2 (20.0)	44.7 (19.9)	43.4 (20.1)	0.2839 [1]
Rales	1095 (94.8%)	679 (94.3%)	416 (95.6%)	0.3249 [2]
Comorbidities				
Hypertension	1006 (86.6%)	602 (83.0%)	404 (92.7%)	<0.0001 [2]
Hyperlipidemia	617 (53.1%)	405 (55.9%)	212 (48.6%)	0.0167 [2]
Diabetes mellitus	551 (47.5%)	347 (47.9%)	204 (46.8%)	0.7229 [2]
Cigarette smoking	153 (13.2%)	127 (17.5%)	26 (6.0%)	<0.0001 [2]
Stroke or other cerebrovascular event	157 (13.5%)	101 (13.9%)	56 (12.8%)	0.5999 [2]
Peripheral vascular disease	155 (13.4%)	115 (15.9%)	40 (9.2%)	0.0012 [2]
Asthma, bronchitis, or COPD	184 (15.8%)	138 (19.0%)	46 (10.6%)	0.0001 [2]
Atrial fibrillation at screening	479 (41.3%)	284 (39.2%)	195 (44.8%)	0.0608 [2]
History of atrial fibrillation or flutter	602 (51.9%)	354 (48.8%)	248 (56.9%)	0.0078 [2]
History of CRT or ICD procedures	294 (25.3%)	218 (30.1%)	76 (17.4%)	<0.0001 [2]
Myocardial infarction	403 (34.7%)	286 (39.4%)	117 (26.8%)	<0.0001 [2]
Depression	60 (5.2%)	34 (4.7%)	26 (6.0%)	0.3425 [2]
Devices				
Pacemaker	121 (10.4%)	70 (9.7%)	51 (11.7%)	0.2701 [2]
Implantable cardiac defibrillator	154 (13.3%)	136 (18.8%)	18 (4.1%)	<0.0001 [2]
Biventricular pacing	113 (9.7%)	96 (13.2%)	17 (3.9%)	<0.0001 [2]
Medication				
ACE inhibitor	633 (54.5%)	392 (54.1%)	241 (55.3%)	0.6894 [2]
ACEi or ARBs	788 (67.9%)	492 (67.9%)	296 (67.9%)	0.9922 [2]
Angiotensin-receptor blocker	185 (15.9%)	112 (15.4%)	73 (16.7%)	0.5594 [2]
Beta-blocker	794 (68.4%)	507 (69.9%)	287 (65.8%)	0.1451 [2]
Aldosterone antagonist	365 (31.4%)	240 (33.1%)	125 (28.7%)	0.1151 [2]
Oral loop diuretic 30 days prior	44.7 (65.2)	50.5 (72.3)	34.9 (49.6)	<0.0001 [S]
Digoxin	228 (19.6%)	116 (16.0%)	112 (25.7%)	<0.0001 [2]
Nitrates at randomization	81 (7.0%)	42 (5.8%)	39 (8.9%)	0.0412 [2]
Baseline laboratory data				
Sodium (mmol/L)	140.82 (3.59)	140.76 (3.60)	140.93 (3.57)	0.4183 [1]
Phosphate (mmol/L)	1.19 (0.32)	1.18 (0.36)	1.20 (0.23)	0.3547 [S]
Calcium (mmol/L)	2.26 (0.15)	2.26 (0.16)	2.27 (0.14)	0.8622 [S]
Hemoglobin (g/dL)	12.79 (1.86)	13.11 (1.89)	12.27 (1.68)	<0.0001 [S]
White blood cell count (×10/L)	8.179 (2.843)	8.022 (2.637)	8.439 (3.142)	0.0243 [S]
Lymphocyte (%)	18.17 (7.81)	18.50 (7.81)	17.64 (7.78)	0.0803 [1]
Potassium (mmol/L)	4.27 (0.63)	4.31 (0.64)	4.21 (0.61)	0.0098 [1]
Creatinine (μmol/L)	116.58 (33.15)	126.28 (32.81)	100.58 (26.94)	<0.0001 [S]
Uric acid (μmol/L)	475.8 (135.9)	488.1 (137.7)	455.4 (130.6)	<0.0001 [1]
Troponin T (μg/L^d^)	0.035 (0.033, 0.037)	0.037 (0.035, 0.040)	0.031 (0.029, 0.034)	0.0015 [S]
BUN (mmol/L)	9.78 (4.03)	10.01 (3.96)	9.40 (4.10)	0.0132 [1]
Cystatin-C (mg/L^d^)	1.45 (1.43, 1.48)	1.46 (1.43, 1.49)	1.44 (1.40, 1.48)	0.4950 [1]
Alanine aminotransferase (U/L^d^)	23.5 (22.7, 24.4)	23.8 (22.7, 25.0)	23.1 (21.8, 24.5)	0.4078 [1]
NT-proBNP (ng/L^d^)	5054 (4795, 5326)	4936 (4615, 5279)	5253 (4830, 5714)	0.2579 [1]
eGFR (mL/min per 1.73 m^2^)	53.49 (13.03)	54.81 (12.84)	51.32 (13.06)	<0.0001 [1]
Total cholesterol (mmol/L)	4.09 (1.17)	3.97 (1.13)	4.30 (1.20)	<0.0001 [1]
Glucose (mmol/L)	7.75 (3.57)	7.62 (3.48)	7.96 (3.71)	0.1202 [1]
Albumin (g/L)	40.23 (4.33)	40.28 (4.57)	40.15 (3.91)	0.6229 [S]

^a^Mean (SD), or geometric mean (95% CI) if log transformed, for continuous variables, *n* (%) for categorical variables (% based on total number of patients with a non-missing value of the characteristic)

^b^Based on *t* test [1], Chi squared test [2], Fisher’s exact test [3], or the Satterthwaite method due to unequal variances in comparison groups [S]

^c^US-Like in the analyses indicates Region 1 vs. Region 2. Region 1 includes patients from United States, France, The Netherlands, Israel, Spain, Germany, Italy, and Poland. Region 2 includes patients from Argentina, Hungary, and Romania

^d^The following baseline laboratory variables have been log transformed: alanine aminotransferase, NT-proBNP, troponin T, cystatin-C

### Symptomatic response by sex

**Fig. 1:**
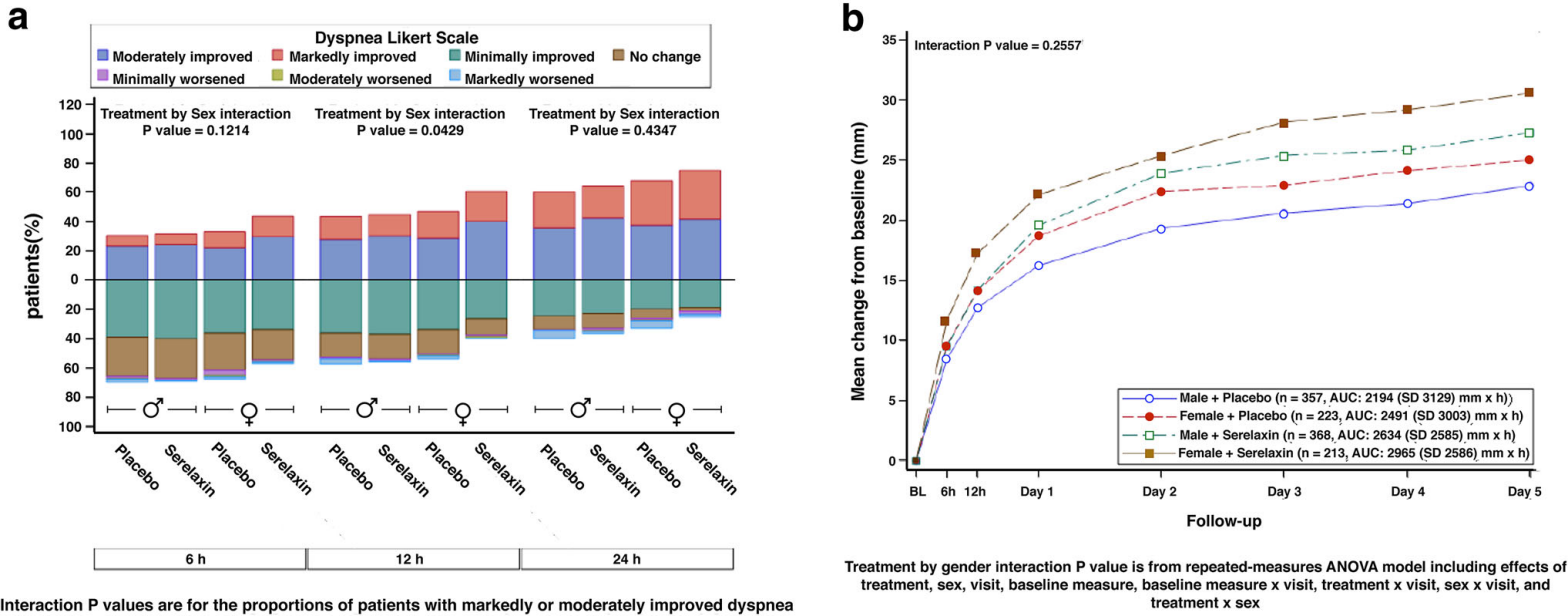
Dyspnea Likert scale (**a**) and VAS AUC change (**b**) by treatment and sex

Details on the symptomatic response by sex are shown in Table 2 and Fig. 1.

**Table 2:** Symptomatic response by sex

	Total cohort^a^ (*n* = 1161)	Men^a^ (*n* = 725)	Women^a^ (*n* = 436)	*p* value^b^
Change from baseline VAS score (mm)				
Hour 6	9.55 (16.51)	8.92 (16.21)	10.60 (16.97)	0.0933 [1]
Hour 12	14.20 (19.33)	13.35 (19.74)	15.63 (18.57)	0.0518 [1]
Day 1	18.74 (23.49)	17.77 (23.35)	20.35 (23.66)	0.0701 [1]
Day 2	22.35 (26.55)	21.46 (27.02)	23.82 (25.70)	0.1420 [1]
Day 5	25.94 (30.77)	24.88 (31.02)	27.68 (30.30)	0.1334 [1]
Day 14	22.72 (34.52)	22.01 (34.56)	23.90 (34.46)	0.3661 [1]
Dyspnea VAS AUC (mm × h)				
Baseline to day 14	7786.82 (9333.49)	7482.07 (9410.64)	8293.56 (9192.12)	0.1515 [1]
Day 1 to day 5	2234.37 (2549.18)	2137.14 (2566.48)	2396.04 (2514.75)	0.0938 [1]
Day 1 to day 14	7489.24 (9077.60)	7201.79 (9152.91)	7967.23 (8940.94)	0.1642 [1]
Dyspnea markedly or moderately improved per Likert scale, *n* (%)				
Hour 6	385 (33.6%)	221 (31.0%)	164 (38.0%)	0.0156 [2]
Hour 12	544 (47.5%)	313 (43.8%)	231 (53.5%)	0.0015 [2]
Day 1	751 (65.5%)	443 (62.0%)	308 (71.1%)	0.0017 [2]
Day 2	850 (74.0%)	510 (71.3%)	340 (78.5%)	0.0070 [2]
Day 5	915 (79.5%)	555 (77.4%)	360 (82.9%)	0.0240 [2]
Day 14	857 (74.4%)	523 (72.8%)	334 (77.0%)	0.1208 [2]
General wellbeing, change from baseline in VAS score (mm)				
Hour 6	9.12 (16.48)	8.63 (16.12)	9.93 (17.06)	0.1933 [1]
Hour 12	13.61 (18.85)	13.04 (18.64)	14.56 (19.19)	0.1843 [1]
Day 1	18.20 (22.72)	17.45 (22.14)	19.44 (23.63)	0.1491 [1]
Day 2	21.19 (25.77)	20.47 (25.86)	22.39 (25.61)	0.2194 [1]
Day 5	24.82 (30.43)	23.50 (30.25)	27.00 (30.64)	0.0579 [1]
Day 14	21.35 (33.67)	20.48 (33.28)	22.81 (34.30)	0.2530 [1]
General wellbeing Likert score				
Hour 6	0.99 (1.11)	0.94 (1.09)	1.07 (1.12)	0.0324 [W]
Hour 12	1.31 (1.17)	1.23 (1.19)	1.44 (1.13)	0.0018 [W]
Day 1	1.60 (1.36)	1.53 (1.36)	1.71 (1.35)	0.0040 [W]
Day 2	1.75 (1.49)	1.64 (1.55)	1.91 (1.38)	0.0012 [W]
Day 5	1.84 (1.77)	1.77 (1.83)	1.97 (1.67)	0.0434 [W]
Day 14	1.56 (2.07)	1.51 (2.11)	1.65 (2.01)	0.3751 [W]

^a^Mean (SD) for continuous variables and *n* (%) for categorical variables (% based on total number of patients with a non-missing value of the characteristic)

^b^Based on *t* tests [1] for continuous variables, and Chi squared tests [2] for categorical variables. Wilcoxon Rank Sum test [W] will be performed for general wellbeing Likert score

The change from baseline in dyspnea visual analog scale AUC to day 5 primary dyspnea endpoint did not vary by sex. However, there was a significantly higher proportion of women than men with moderate or marked dyspnea improvement measured by Likert scale during the first 24 h, which was the other primary endpoint in RELAX-AHF.

Likewise, the change from baseline in dyspnea visual analog scale score was not different in women compared to men. But there were consistently higher rates of women with markedly or moderately improved dyspnea per Likert scale and higher general wellbeing Likert score values in women, both through 24 h and through 5 days, respectively.

### Diuretic doses, treatment response and post-discharge outcome by sex

**Fig. 2:**
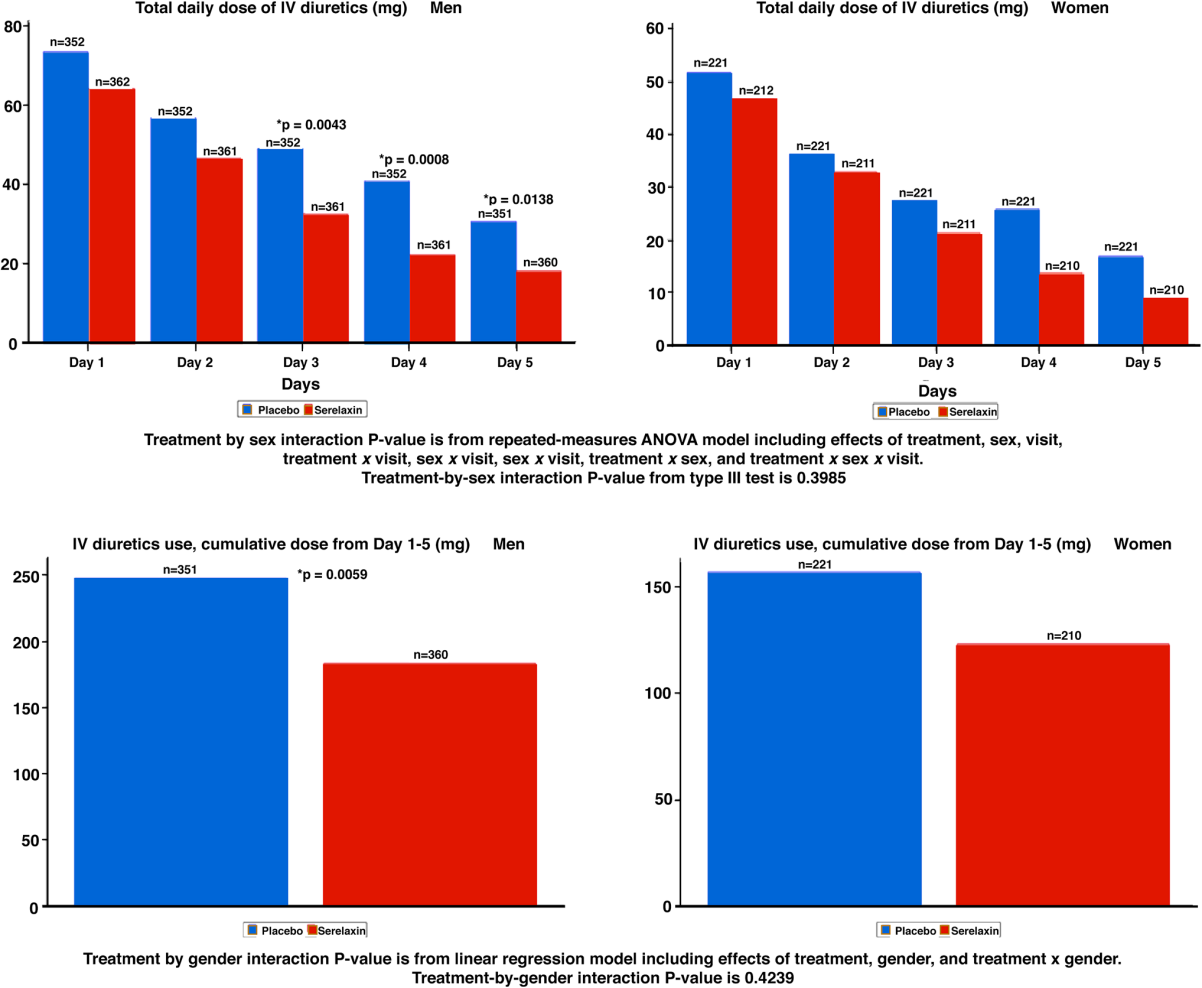
Total daily dose of IV diuretics from day 1 to day 5 stratified by treatment and sex

**Fig. 3:**
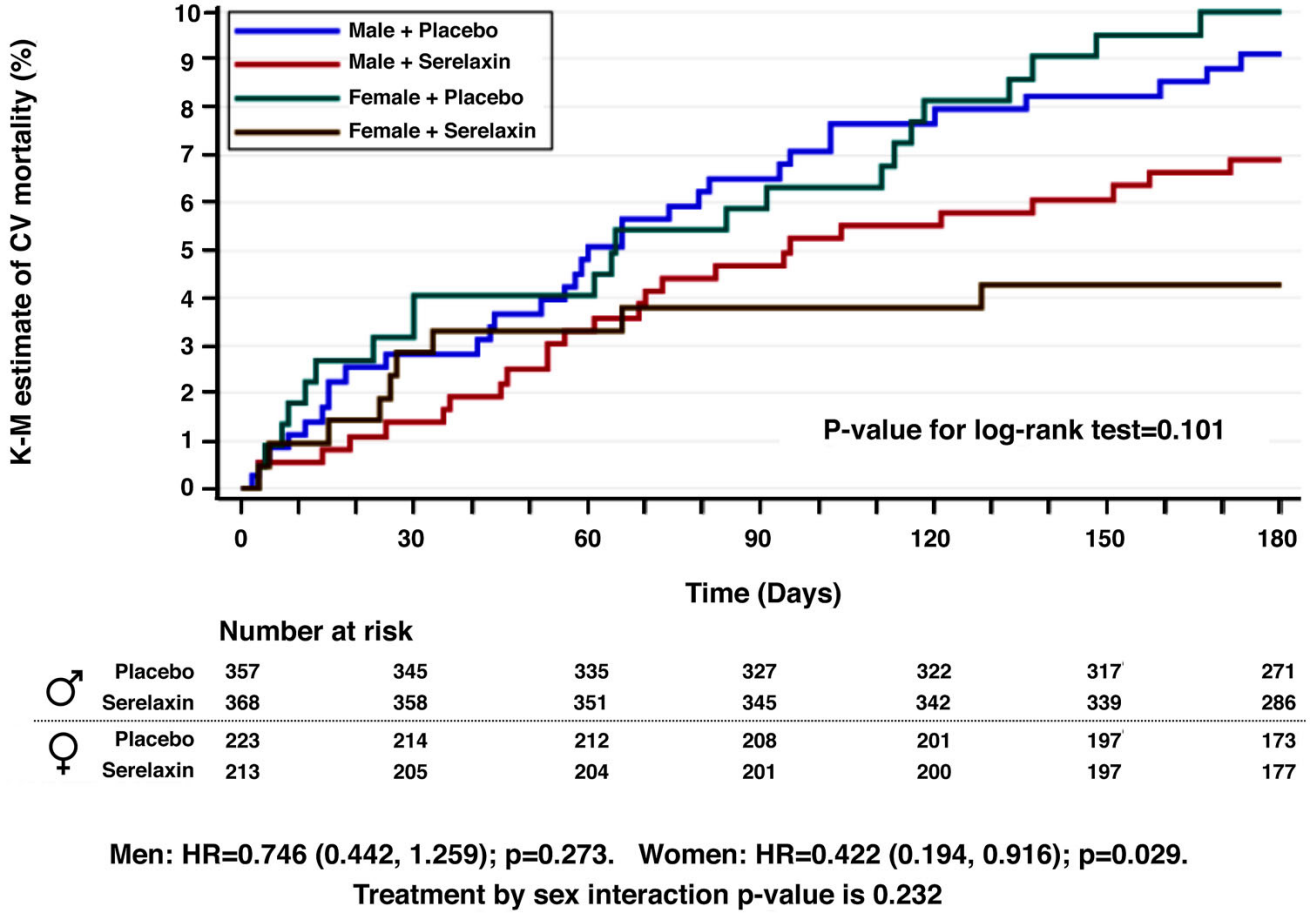
Kaplan–Meier estimates for 180-day cardio-vascular mortality with numbers at risk by treatment and sex

Details on the diuretic doses, treatment response and post-discharge outcome by sex are shown in Table 3. Women were treated with lower total IV and oral loop diuretic doses through day 5, respectively (Fig. 2), but dyspnea improved earlier moderately or markedly through day 5 in women. There were no relevant sex differences regarding changes in neither body weight nor relative body weight, worsening heart failure and outcome (Fig. 3), but women showed a trend towards longer ICU/CCU stays [mean (SD), 4.05 (7.67) days vs. 3.51 (6.63) days, *p* = 0.0248] and total initial hospital stays [10.37 (9.62) days vs. 9.87 (9.17) days, *p* = 0.0258] compared to men.

**Table 3:** Inotrope/vasoactive medication, diuretic doses, treatment response and post-discharge outcome by sex

	Total cohort^a^ (*n* = 1161)	Men^a^ (*n* = 725)	Women^a^ (*n* = 436)	*p* value^b^
Inotrope/vasoactive medication				
All IV inotrope/vasoactive agents through day 5	161 (13.9)	93 (12.8)	68 (15.6)	0.1862 [C]
Nitroglycerin	127 (10.9)	66 (9.1)	61 (14.0)	0.0098 [C]
Diuretic doses and treatment response				
Total IV loop diuretic dose through day 5 (mg)	187.21 (316.02)	215.36 (364.91)	140.76 (204.10)	<0.0001 [S]
Total oral loop diuretic dose through day 5 (mg)	187.70 (191.79)	199.12 (204.05)	168.86 (168.20)	0.0067 [S]
Treatment response				
Study day of moderately or markedly improved dyspnea through day 5	1.72 (2.00)	1.87 (2.07)	1.48 (1.85)	0.0005 [W]
Study day of worsening HF through day 5	5.65 (1.19)	5.61 (1.25)	5.71 (1.08)	0.1395 [W]
Worsening HF through day 14	157 (13.56%)	106 (14.67%)	51 (11.71%)	0.1522 [L]
Change in bodyweight from baseline (kg)				
Day 1	−1.48 (1.98)	−1.56 (2.11)	−1.33 (1.73)	0.0433 [S]
Day 2	−2.03 (2.46)	−2.13 (2.65)	−1.87 (2.09)	0.0752 [S]
Day 5	−2.86 (3.34)	−2.93 (3.50)	−2.76 (3.05)	0.4210 [S]
Day 14	−3.31 (4.26)	−3.49 (4.60)	−3.02 (3.64)	0.0642 [S]
Relative change in bodyweight from baseline (%)				
Day 1	−1.82 (2.38)	−1.83 (2.44)	−1.81 (2.28)	0.8637 [1]
Day 2	−2.48 (2.90)	−2.45 (2.97)	−2.53 (2.80)	0.6483 [1]
Day 5	−3.46 (3.91)	−3.30 (3.84)	−3.72 (4.01)	0.0797 [1]
Day 14	−3.87 (4.75)	−3.82 (4.89)	−3.95 (4.53)	0.6780 [1]
Outcome				
Uncontrolled blood pressure^c^	271 (23.3%)	157 (21.7%)	114 (26.1%)	0.0798 [C]
Length of ICU/CCU stay (days)	3.71 (7.04)	3.51 (6.63)	4.05 (7.67)	0.0248 [W]
Length of initial hospital stay (days)	10.06 (9.34)	9.87 (9.17)	10.37 (9.62)	0.0258 [W]
Days alive and out of hospital before day 30	20.61 (6.64)	20.68 (6.80)	20.47 (6.38)	0.1001 [W]
Death or worsening HF or readmission to hospital for HF through day 30	200 (17.30%)	129 (17.87%)	71 (16.34%)	0.4556 [L]
CV death or readmission to hospital for HF or renal failure through day 30	83 (7.20%)	50 (6.95%)	33 (7.62%)	0.6769 [L]
CV death or readmission to hospital for HF or renal failure through day 30 after discharge	92 (8.17%)	55 (7.82%)	37 (8.76%)	0.5790 [L]
All-cause death through day 30	31 (2.68%)	16 (2.22%)	15 (3.46%)	0.2100 [L]
All-cause death or readmission to hospital for HF or renal failure through day 60	154 (13.36%)	97 (13.48%)	57 (13.17%)	0.9028 [L]
CV death through day 180	88 (7.68%)	57 (7.98%)	31 (7.20%)	0.6484 [L]
All-cause death through day 180	107 (9.31%)	68 (9.46%)	39 (9.06%)	0.8059 [L]

^a^Mean (SD) for continuous variables; *n* (%) for categorical variables (% based on total number of patients with a non-missing value of the characteristic); and number of events (K-M%) for time-to-event variables

^b^Based on Satterthwaite method [S] (if equal variance assumption violated) for continuous variables, Wilcoxon rank sum test [W] for count variables, Chi squared test [C] for categorical variables, and logrank test [L] for time-to-event variables

^c^Uncontrolled blood pressure is defined as systolic >150 mmHg or diastolic >90 mmHg at day 2 and through day 5, or at time of discharge (if discharge time ≤day 5), whichever occurs first

### Interaction analyses of differential effects by sex

**Fig. 4:**
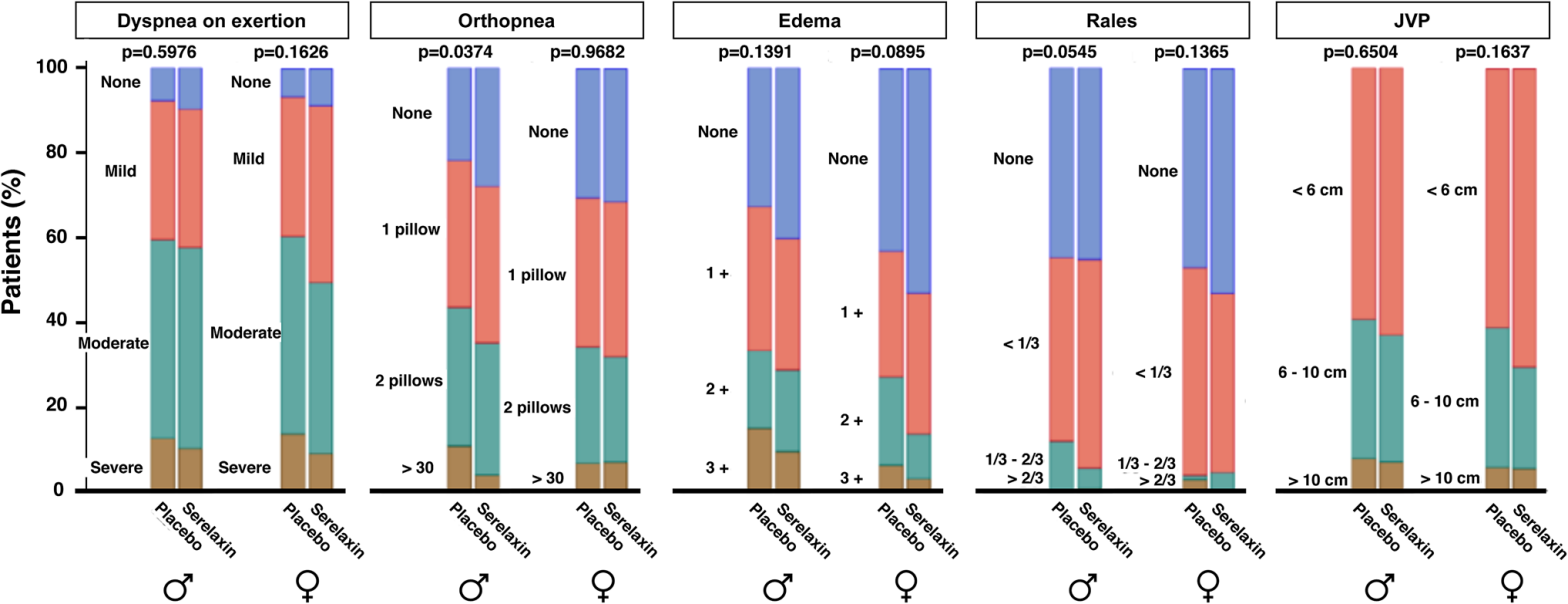
Physician-assessed signs and symptoms of congestion at day 2 by treatment and sex

Physician-assessed signs and symptoms of congestion, such as dyspnea on exertion, orthopnea, edema or rales, did not vary by treatment and sex (Fig. 4). Moreover, women did not show different outcome than men in any of the analyzed endpoints. Neither did the relationship of sex with outcome vary by treatment with serelaxin, the presence of heart failure with preserved ejection fraction or both characteristics.

### Effect of selected characteristics on early dyspnea improvement

A multi-variable logistic regression model was built to assess the association between moderately or markedly better dyspnea on the Likert scale at 6, 12 and 24 h using selected baseline characteristics, such as sex, HF status, age, pulse pressure, heart rate, and loop diuretic dose as covariates (Table 4). Male sex, age and total diuretic dose showed independent negative association with dyspnea improvement within 24 h.

**Table 4:** Association between moderately or markedly better dyspnea on the Likert scale at 6, 12 and 24 h and selected characteristics (*n* = 1076)

Covariates	Coefficient (95% CI)	Std. err	Odds ratio (95% CI)	*p* value
Male	−0.5032 (−0.8053, −0.2011)	0.1541	0.605 (0.447, 0.818)	0.0011
HFpEF (LVEF ≥50)	−0.1579 (−0.4959, 0.1801)	0.1725	0.854 (0.609, 1.197)	0.3598
Age	−0.0200 (−0.0330, −0.0070)	0.0066	0.980 (0.968, 0.993)	0.0026
Pulse pressure	−0.0025 (−0.0113, 0.0064)	0.0045	0.998 (0.989, 1.006)	0.5861
Heart rate	0.0055 (−0.0041, 0.0152)	0.0049	1.006 (0.996, 1.015)	0.2607
Total diuretic dose^a^	−0.0031 (−0.0053, −0.0009)	0.0011	0.997 (0.995, 0.999)	0.0067

Logistic regression model to estimate the relationship between the dependent variable and the covariates

^a^Total diuretic dose is defined as IV loop diuretics dose + 0.5 × oral loop diuretics on day 1

### Changes in biomarkers

**Fig. 5:**
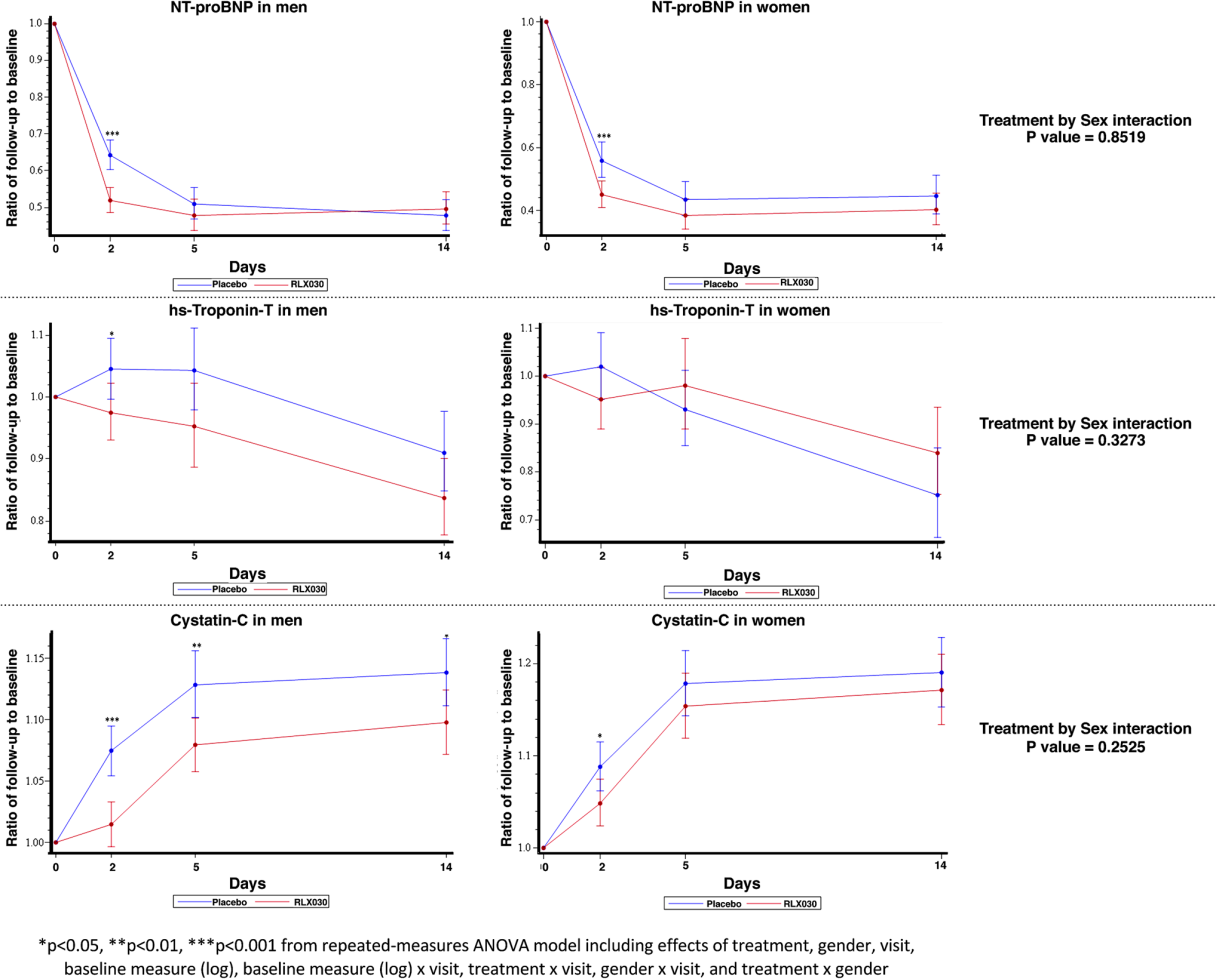
Changes in hs-troponin-T, NT-proBNP, and cystatin-C by treatment and sex

The changes of hs-troponin-T, NT-proBNP, and cystatin-C from baseline to day 2, 5, and 14 in men and women are presented in Fig. 5. Both, men and women, showed a marked early decrease of NT-proBNP levels during the first 2 days of treatment. These levels further decreased in both sexes through day 5 and persisted through day 14. NT-proBNP changes were paralleled by increasing levels of cystatin-C in both sexes by day 5 and day 14. The levels of hs-troponin-T decreased through days 5 and 14 in men and women.

## Discussion

Marked or moderate early dyspnea improvement using the 7-item Likert scale was seen more often in women admitted for acute heart failure within the first 24 h, and persisted over the first 5 days. These improvements were not paralleled by the VAS-scale assessments. Similarly, general wellbeing, using the Likert scale over the first 5 days, improved significantly better in women, but this was not seen when the VAS wellbeing score was used. Other clinical outcome parameters were similar between men and women, and serelaxin was equally effective in men and women.

### Dyspnea improvement by sex

Dyspnea is a complex symptom in patients with acute heart failure. Patient-reported dyspnea relief is a clinically meaningful treatment goal because persistent dyspnea is associated with adverse outcome [9, 10]. It is advocated as a patient-relevant endpoint for heart failure trials by regulatory authorities [11].

Therefore, our findings that women have a greater early dyspnea relief may be of clinical relevance. Although this difference could likely be explained by factors related to female sex, such as hypertension and heart failure with preserved ejection fraction (HFpEF), our multivariable analysis showed that female sex was an independent predictor of early dyspnea relief. No sex differences in dyspnea relief between men and women were found in a post hoc analysis of the PROTECT pilot study. However, patient numbers were small and no adjustments for preserved left ventricular ejection fraction were made [12]. Comparable studies of early dyspnea relief in acute heart failure patients have not considered sex as covariate [10].

The underlying cause of our findings is likely multifactorial. Remarkably, more intense dyspnea relief in women occurred despite using lower total IV and oral loop diuretic doses and less weight loss through day 5 in women compared to men, and comparable drops of NT-proBNP values were seen in both sexes. These findings can be explained pathophysiologically by a typical mechanism of cardiac decompensation in women, which is frequently caused by fluid redistribution and related to the higher proportion of HFpEF and hypertension among women [13]. In this context, the slightly higher proportion of women being treated with intravenous nitrates during the first days of treatment could have favored the faster improvement of dyspnea in women. However, the absolute numbers of patients who were treated with intravenous inotrope/vasoactive medication were relatively low and it appears unlikely that this fully explains the differential dyspnea response by sex.

Theoretically, the higher rates of concomitant pulmonary disease, such as asthma, bronchitis, or COPD, in men, might have negatively affected dyspnea response in men. However, patients with relevant severity of these diseases were not included in the trial based on the exclusion criteria. Also, less diuretic treatment and less weight loss could be required in women to reach comparable outcome and better symptomatic relief. It is evidenced by trial results and registry data in acute heart failure that the overall outcome does not differ between men and women despite significantly lower diuretic doses and less absolute weight loss [3, 14]. It should be noted that all measured surrogates of congestion at baseline, such as edema, orthopnea, jugular venous pressure, dyspnea on exertion, and rales, were well balanced between men and women. Thus, it might be speculated that different symptom perception per se is responsible for the higher rates of moderate to marked dyspnea relief in women over men. Alternatively, underlying pathophysiological differences in fluid distribution may have translated to differences in symptoms. An acute cardiovascular, hypertensive type of failure has been proposed as subtype of acute heart failure [13]. It is common in the elderly, patients with history of hypertension, and women, and is characterized by preserved left ventricular ejection fraction [13]. In this type of heart failure, ventricular and vascular stiffness play important pathophysiological roles, with a vulnerability to any disorder of fluid balance, resulting in a rapid increase of vascular resistance, blood pressure and increased pulmonary pressure [13]. Notably, the increase in pulmonary arterial pressure and the pulmonary capillary wedge pressure was previously identified as major determinants of dyspnea in an analysis of the hemodynamics of acute heart failure patients [15]. Because data from community dwelling subjects have proven that women have greater aortic stiffening and lower total arterial compliance than men [16], it might be speculated that rapid changes of intracardiac and intrapulmonary pressures as surrogates for dyspnea depend, to a greater extent, on changes of vascular volume load in women than in men. This could explain why early vasoactive and decongestive therapy might have more intense effects in women than in men, and that this could translate into differences in perceived dyspnea relief. These effects are more likely to be pronounced early during standard treatment, when the most intense reduction of vascular resistance and ventricular afterload is usually seen in acute heart failure patients [15].

The question why marked or moderate early dyspnea improvement was seen more often in women using the 7-item Likert scale and not by the VAS-scale assessments could be due to the different aspects that are covered by the two different measurement scales. Notably, the Likert scale was used to assess early (6–24 h) dyspnea relief, whereas the VAS-scale was used to quantify persistent dyspnea relief by the change in VAS area under the curve (VAS AUC) through day 5, as specified in the study protocol [17]. The MEASURE-HF study indicated that the Likert scale categories reflect initial improvement relative to the baseline status without capturing relevant improvement at later timepoints; contrarily, VAS scores of dyspnea improved steadily [18]. Likert scale assessment emphasizes the change of perceived dyspnea compared to the most severe symptom sensation at baseline. The effect is more clearly measurable as an improvement on its categorical scale with higher initial symptom severity, but it is increasingly difficult to be captured as more time passes by. VAS scores capture the particular instantaneous state of dyspnea on a continuous scale individually at different timepoints. However, it is noteworthy that a trend towards higher change from baseline VAS score (mm) in women compared to men was detectable at the time points 6 and 12 h and after 1 day (*p* = 0.0933, *p* = 0.0518 and *p* = 0.0701, respectively) which also translated to a trend toward higher change from dyspnea VAS AUC (mm × h) from day 1 to day 5 (*p* = 0.0938) in women.

### Serelaxin effects in men and women

Although relaxin is commonly known to exert various biologic effects in women during pregnancy [19, 20], data on sex-specific cardio-vascular effects are scarce. Debrah et al. demonstrated in a rat model that recombinant relaxin increases cardiac output and reduces arterial load in both male and female rats [21]. In a recent subgroup analysis, it was shown that serelaxin was equally effective in men and women [22]. The results of our interaction analyses confirm that sex does not modify the effects of serelaxin on dyspnea relief and/or any death- or rehospitalization-related outcomes in men and women.

### Limitations

All limitations of retrospective subgroup analyses apply to our study. No hypotheses regarding sex differences have been pre-specified, the ratio of men to women is unbalanced and, thus, our results could be biased. All results only have a hypothesis-generating character. The great majority of the women were white (i.e., 95%), so that results are not generalizable to black women, an increasing proportion of the heart failure population with high likelihood for poor outcomes. Also, validation of the VAS and the Likert scale in acute heart failure is largely based on data from men and lacking sex-specific validation.

## Conclusion

Women exhibit better earlier dyspnea relief and improvement in general wellbeing as compared with men, even adjusted for age and left ventricular ejection fraction. However, in-hospital and post-discharge clinical outcomes were similar between men and women.

## Appendix

See Tables 5 and 6.

**Table 5:** Interaction analysis of sex and treatment interaction (unadjusted) (*n* = 1161)

Outcome	*p* value for interaction^a^ sex by treatment
All-cause death through day 180	0.7229
CV death through day 60	0.7151
Days alive and out of hospital through day 60	0.2473
CV death or HF/RF rehospitalization through day 60	0.7247
RF rehospitalization through day 60	0.9933
HF rehospitalization through day 60	0.8778
Worsening heart failure through day 5	0.6764
SCr increase of ≥0.3 mg/dL above baseline through day 5	0.0585
Dyspnea VAS AUC to day 5	0.9228
Time to moderately or markedly better dyspnea through day 5 (days)	0.2050
Dyspnea Likert scale moderately/marked better at 6, 12, and 24 h	0.3869

^a^Interaction *p* value comes from multiple linear regression models for continuous outcomes, logistic regression models for categorical outcomes, Cox proportional hazards models for time-to-event outcomes

**Table 6:** Interaction analysis of sex, treatment, and HF interaction (unadjusted) (*n* = 1091)

Outcome	*p* value for interaction^a^ sex by treatment	*p* value for interaction^a^ sex by HFpEF	*p* value for interaction^a^ HFpEF by treatment	*p* value for interaction^a^ sex by treatment by HF
Dyspnea VAS AUC to day 5	0.4714	0.4987	0.2436	0.0945
Dyspnea Likert scale moderately/marked better at 6, 12, and 24 h	0.6301	0.1481	0.5462	0.2927
Days alive and out of hospital through day 60	0.0790	0.0737	0.0488	0.0737
CV death or HF/RF rehospitalization through day 60	0.7925	0.5682	0.9335	0.9726
CV death through day 60	0.2961	0.1565	0.2685	0.1468
HF rehospitalization through day 60	0.5582	0.9552	0.5330	0.4230
RF rehospitalization through day 60	0.9947	1.0000	0.9970	0.9999
All-cause death through day 180	0.9661	0.3898	0.7160	0.7908
Time to moderately or markedly better dyspnea through day 5 (days)	0.7899	0.5204	0.2896	0.1543
SCr increase of ≥0.3 mg/dL above baseline through day 5	0.0602	0.4721	0.8447	0.7312
Worsening heart failure through day 5	0.4598	0.3090	0.0864	0.0319

HFpEF vs. HFrEF

Subjects with missing HF were omitted from the analysis

^a^Interaction *p* value comes from multiple linear regression models for continuous outcomes, logistic regression models for categorical outcomes, Cox proportional hazards models for time-to-event outcomes
